# Dietary calcium, magnesium, and phosphorus intakes and risk of stroke in Chinese adults

**DOI:** 10.1038/s41598-021-90388-z

**Published:** 2021-05-28

**Authors:** Hai-Lu Zhu, Yan Liu, Jian Zhang, Ming-Xu Wang, Hong Jiang, Fang Guo, Ming Li, Fei-Fei Qi, Xiao-Hong Liu, Le Ma

**Affiliations:** 1 The First Affiliated Hospital, Xi’an Jiaotong University Health Science Center, Xi’an 710061, People’s Republic of China; 2 Key Laboratory of Shaanxi Province for Craniofacial Precision Medicine Research, College of Stomatology, Xi’an Jiaotong University, Xi’an, China; 3 School of Public Health, Xi’an Jiaotong University Health Science Center, Xi’an 710061, People’s Republic of China; 4 School of Public Health, University of Hong Kong, Hong Kong Special Administrative Region 999077, Pok Fu Lam, People’s Republic of China; 5 Center for Population Health Research, Division of Health Sciences, University of South Australia, Adelaide, South Australia 5000 Australia

**Keywords:** Diseases, Risk factors

## Abstract

Controversial results have been reported about the association of calcium, magnesium, and phosphorus and stroke risk, but none in China. To investigate the association between dietary calcium, magnesium, phosphorus, and stroke incidence in Chinese adults, we collected data from the China Health and Nutrition Survey (CHNS) from 2004 to 2011, including 6411 participants aged 45–79 years and free of stroke at baseline. Diet was assessed by interviews combining 3-d 24-h food recalls and household food inventory weighing at each survey round. The stroke incident was identified based on the validated self-report. Multivariate Cox regression models were used to estimate hazard ratios (HRs) and 95% confidence intervals (CIs). For 32,024 person-years of follow-up, 179 stroke cases were documented. After adjustment for major lifestyle and dietary risk factors, calcium intake was positively associated with reduced stroke risk, and the HR of stroke comparing extreme quartiles was 0.53 (95% CI 0.29–0.96, *P*_trend_ = 0.03). In further stratified analyses, significant heterogeneity across sex strata was found (*P*_interaction_ = 0.03). Dietary calcium intake among men was more inversely related to stroke, with HRs being 0.33 (95% CI 0.15–0.76, *P*_trend_ = 0.02), compared to 1.24 (95% CI 0.46–3.35, *P*_trend_ = 0.89) among women. However, no significant association between stroke and magnesium or phosphorus was revealed. Our findings suggest that higher dietary calcium intake was associated with a lower risk of stroke in Chinese adults, particularly in men.

## Introduction

As a cerebrovascular disease, stroke has become the leading cause of death in developed and developing countries. A 10% overall annual death in the world is estimated to be attributable to it^1^. Even the stroke survivors, approximately 15–30% are permanently disabled^2^, which results in approximately 113 million disability-adjusted life years lost^3^. China has the highest estimated lifetime risk of stroke worldwide (39.3%) according to the 2016 global burden of disease (GBD) regional study^4^. Apparently, stroke has become a significant public health issue for China in facing the increased aging population. Therefore, identifying the modifiable risk factors is pressing for the prevention and the improvement of life quality of stroke patients and the reduction of massive socio-economic burden.

Although the exact etiology is not entirely elucidated, stroke is generally considered a disease related to genetic and environmental risk factors (especially dietary) or the interplay of these factors. Extensive evidence suggests that dietary modifications are beneficial to prevent the onset of stroke^5–7^. A close relationship exists between calcium, magnesium, phosphorus, and vascular protection, which are considered important health implications for vascular diseases since they can regulate vascular calcification and endothelial dysfunction^8,9^. Up to now, several studies have examined the relationship between these nutrients intake and stroke risk^10–12^, but the results are inconsistent. For example, research in the European population showed magnesium intake, but not calcium was significantly related to a lower risk of stroke^11^. In contrast, in the United States, calcium intake, instead of magnesium, leads to decrease stroke hazard in women, while among men, magnesium intake is inversely associated with stroke^12^. Furthermore, most of these researches were conducted in western populations with high intakes of these minerals^13,14^. However, to our knowledge, little is known about the association of dietary calcium, magnesium, and phosphorus intakes with stroke incidence in the Chinese population. The average dietary intake of these micronutrients among Chinese individuals consuming predominantly plant-based diets was reported to be lower than that of Europeans and Americans who have more dairy and meat products^15^. In this context, a large-scale prospective study in the Chinese population to supplement the association's evidence is admittedly needed. We, therefore, used data from the China Health and Nutrition Survey (CHNS) to evaluate the relationship between dietary calcium, magnesium, and phosphorus intakes and subsequent incidence of stroke in Chinese adults.

## Results

During 32,024 person-years of follow-up among the 6411 participants (mean age 56 years and 48.2% men), 179 cases (117 men) of incident stroke were identified. According to the quartile of dietary calcium, magnesium, and phosphorus intake, the baseline characteristics of the participants were shown in Table 1. After the normality test, we found that some variables do not satisfy the normal distribution (SFA, PUFA). We used the non-parametric test to examine differences and trends between groups, the t-test for the continuous variables with normal distribution, and the chi-square test for the classified variables. Participants with a higher intake of these minerals had higher levels of education and individual incomes. Higher proportions were observed in participants with high calcium intakes than those with lower calcium intakes of smoking and alcohol consumption (39.4% vs. 29.3%; 42.3 vs. 24.2%, respectively). Besides, they tended to be more physically active and consumed more cereal fiber and cholesterol. Subjects with a higher magnesium and phosphorus consumption confirmed nearly comparable traits to individuals with a higher calcium intake. Based on Spearman correlations, calcium intake was strongly positively associated with magnesium intake (r = 0.64, *P* < 0.001) and phosphorus intake (r = 0.65, *P* < 0.001). Intakes of magnesium and phosphorus were also highly associated (r = 0.85, *P* < 0.001). The relationship between intakes of calcium, magnesium, phosphorus, and stroke risk was presented in Table 2. Dietary calcium consumption was significantly associated with a lower stroke threat in the age- and sex-adjusted model. After additional adjustment for sociodemographic and lifestyle factors (Model 2), this association was slightly strengthened with an HR of 0.59 (95% CI 0.37–0.94, *P* for trend = 0.02) comparing the lowest quartile with the highest quartile. Further adjustment for potential dietary risk (Model 3) still adhered to this association: the multivariate relative risk in the highest, as opposed to the lowest quartile of calcium consumption, was 0.53 (95% CI 0.29–0.96, *P* for trend = 0.03).

**Table 1:** Baseline characteristics of participants according to quartiles of dietary calcium, magnesium, and phosphorus intakes

Characteristics^1^	Total	Calcium intake		P	Magnesium intake		P	Phosphorus intake		P
		Q1	Q4		Q1	Q4		Q1	Q4	
No. of subjects (n)	6411	1602	1603		1602	1603		1603	1603	
Age at cohort entry (years)	56.0	57.0	55.9	< 0.001	57.9	55.1	< 0.001	58.1	54.6	< 0.001
Man (%)	48.2	39.4	55.1	< 0.001	34.8	62.4	< 0.001	32.6	64.9	< 0.001
Urbanization Index (%)				< 0.001			< 0.001			0.01
Low	33.3	39.1	23.6		27.2	39.8		32.7	35.3	
Medium	33.3	33.8	31.7		31.7	36.5		32.6	34.0	
High	33.4	27.1	44.7		41.2	23.6		34.7	30.7	
Education attained (%)				< 0.001			0.66			< 0.001
Primary	77.7	82.9	71.2		78.4	79.3		81.8	74.1	
Secondary	13.2	11.2	15.1		13.2	13.7		11.3	15.4	
College/University	9.1	5.9	13.7		8.4	9.2		6.9	10.5	
Household income (%)				< 0.001			0.07			< 0.001
Low	44.5	53.9	32.8		47.1	41.8		50.8	37.2	
Middle	33.6	29.3	37.6		31.0	36.6		31.5	36.6	
High	21.9	16.8	29.6		22.0	21.6		17.7	26.2	
Smoker (%)	34.6	29.3	39.4	< 0.001	24.7	44.4	< 0.001	25.1	45.6	< 0.001
Alcohol consumption (%)	34.1	24.2	42.3	< 0.001	23.2	45.5	< 0.001	22.0	47.3	< 0.001
Physical activity level (%)				< 0.001			< 0.001			< 0.001
Light	31.3	40.5	29.3		42.1	29.4		42.4	27.6	
Moderate	35.3	29.6	37.9		33.9	30.6		32.3	32.3	
Vigorous	33.4	29.9	32.8		24.0	39.9		25.3	40.0	
BMI (kg/m^2^)	23.6	23.1	23.9	< 0.001	23.3	23.6	0.03	23.2	23.8	< 0.001
Hypertension (%)	38.5	40.6	38.8	0.31	42.4	35.1	< 0.001	40.9	36.3	0.01
Diabetes (%)	2.6	3.0	2.4	0.04	2.2	2.2	0.99	2.7	2.4	0.82
Myocardial infarction (%)	0.5	0.8	0.5	0.27	0.9	0.4	0.07	0.9	0.3	0.04
Medication use (%)	9.5	8.2	11.3	0.01	11.6	8.2	0.01	10.8	8.7	0.04
Nutrient intake										
Energy (kcal/d)	2220.9	1830.8	2544.9	< 0.001	1694.7	2768.5	< 0.001	1638.4	2832.6	< 0.001
Whole grain (g/d)	403.8	356.2	420.8	< 0.001	313.5	504.4	< 0.001	310.7	509.8	< 0.001
Red meat (g/d)	71.9	58.1	86.0	< 0.001	61.6	74.1	< 0.001	46.7	90.6	< 0.001
fruits (g/d)	43.7	25.9	69.5	< 0.001	29.6	51.9	< 0.001	30.9	48.7	< 0.001
vegetables (g/d)	347.8	267.7	431.2	< 0.001	267.9	420.5	< 0.001	272.9	419.5	< 0.001
SFA (g/d)	9.6	8.0	11.7	< 0.001	8.4	11.0	< 0.001	7.7	11.4	< 0.001
PUFA (g/d)	16.9	13.3	20.8	< 0.001	13.5	20.7	< 0.001	13.3	20.6	< 0.001
Cereal fiber (g/d)	12.0	8.0	15.6	< 0.001	7.6	18.0	< 0.001	8.0	17.5	< 0.001
Na (mg/d)	5413.2	4349.7	6434.1	< 0.001	4305.1	6709.1	< 0.001	4510.1	6358.2	< 0.001
K (mg/d)	1690.7	1258.9	2233.6	< 0.001	1178.6	2312.1	< 0.001	1169.9	2318.5	< 0.001
Cholesterol (g/d)	261.4	198.9	342.4	< 0.001	226.5	286.1	< 0.001	180.8	328.3	< 0.001

BMI, body mass index; SFA, Saturated fatty acid; PUFA, Polyunsaturated fatty acids.

^1^Data are expressed as mean values for continuous variables or percentage (%) for categorical variables.

The t-test is used for the continuous variables with normal distribution. The non-parametric test is applied to the continuous variables with non-normal distribution, and the chi-square test is used for the classified variables.

**Table 2:** Hazard ratios (95% confidence intervals) of stroke according to quartiles of dietary calcium, magnesium, and phosphorus intakes

	Dietary nutrition intake				*P*_for trend_^1^
	Q 1 (low)	Q 2	Q 3	Q 4 (high)	
*Calcium*					
Median intake (mg/d)	222	321	413	677	
Person-years	5748	6705	6770	6021	
Stroke cases (n)	52	51	43	33	
Model 1	1.00	0.87 (0.59, 1.28)	0.72 (0.48, 1.08)	0.59 (0.38, 0.92)	0.01
Model 2	1.00	0.90 (0.60, 1.35)	0.65 (0.42, 1.02)	0.59 (0.37, 0.94)	0.02
Model 3	1.00	0.87 (0.57, 1.34)	0.66 (0.41, 1.07)	0.53 (0.29, 0.96)	0.03
*Magnesium*					
Median intake (mg/d)	200	265	320	443	
Person-years	5466	6625	6798	6356	
Stroke cases (n)	53	39	41	46	
Model 1	1.00	0.66 (0.43, 1.00)	0.70 (0.46, 1.06)	0.77 (0.51, 1.17)	0.43
Model 2	1.00	0.69 (0.44, 1.07)	0.72 (0.46, 1.12)	0.96 (0.62, 1.48)	0.85
Model 3	1.00	0.76 (0.47, 1.21)	0.78 (0.47, 1.31)	0.97 (0.51, 1.85)	0.90
*Phosphorus*					
Median intake (mg/d)	667	875	1043	1393	
Person-years	5732	6618	6722	6172	
Stroke cases (n)	61	46	28	44	
Model 1	1.00	0.70 (0.48, 1.04)	0.41 (0.26, 0.64)	0.67 (0.45, 1.01)	0.05
Model 2	1.00	0.75 (0.49, 1.12)	0.41 (0.25, 0.66)	0.80 (0.52, 1.24)	0.26
Model 3	1.00	0.84 (0.53, 1.31)	0.47 (0.26, 0.85)	0.92 (0.41, 2.03)	0.82

^1^Tests for trend were conducted by modeling the median of each quartile-defined category as a continuous variable in Cox proportional hazards models.

Model 1, adjusted for age and sex.

Model 2, further adjusted for urbanization index, education, household income, smoking status, alcohol intake, physical activity, BMI, hypertension, diabetes, myocardial infarction, and medication use based on model 1.

Model 3, further adjusted for energy, whole grain, red meat, fruits, vegetables, saturated fat, polyunsaturated fat, cereal fiber, Na, K, and cholesterol intakes based on model 2.

No significant association was found between dietary magnesium intake and stroke risk in all three models. In full-model adjustment, the multivariable HR based on comparing the highest and lowest quartile of dietary magnesium consumption changed into 0.97 (95% CI 0.51–1.85, *P* for trend = 0.90). Similarly, phosphorus intakes were not significantly associated with the stroke risk (HR: 0.92, 95% CI 0.41–2.03, *P* for trend = 0.82) by using the same adjustment.

Table 3 shows stratified analyses of relative risks of stroke in line with quartiles of dietary calcium consumption. The inverse association of calcium intake with stroke development was mostly unchanged among participants with various risk profiles characterized by smoking, drinking, BMI, and hypertension status (all *P*_for interaction_ > 0.10). However, the association between calcium consumption and stroke risk became drastically changed by sex (*P*_for interaction_ = 0.03). The multivariate-adjusted HR of stroke for the highest vs. the lowest quintile of calcium intake was 0.33 (95% CI 0.15–0.76, *P*_for trend_ = 0.02) among man and 1.24 (95% CI 0.46–3.35, *P*_for trend_ = 0.89) among women. Likewise, we also observed a statistically significant interaction between dietary calcium consumption and age in relation to the danger of stroke (*P*_for interaction_ = 0.06), the inverse association for calcium consumption seemed more potent for participants who were more youthful than 60 years, compared with those 60 years and older.

**Table 3:** Stratified hazard ratios (95% confidence intervals)^1^ of stroke according to quartiles of dietary calcium intake by various characteristics of participants.

	Dietary calcium intake				*P*_for trend_^2^	*P*_for interaction_^3^
	Q 1 (low)	Q 2	Q 3	Q 4 (high)		
*Sex*^*4*^						
Man	1.00	0.53 (0.31, 0.91)	0.53 (0.29, 0.97)	0.33 (0.15, 0.76)	0.02	0.03
Women	1.00	1.95 (0.92, 4.14)	0.93 (0.38, 2.35)	1.24 (0.46, 3.35)	0.89	
*Age (years)*						
< 60	1.00	0.68 (0.37, 1.27)	0.59 (0.28, 1.24)	0.28 (0.09, 0.80)	0.02	0.06
≥ 60	1.00	1.09 (0.59, 2.00)	0.73 (0.36, 1.48)	0.90 (0.39, 2.06)	0.66	
*Cigarette smoking*						
Yes	1.00	0.82 (0.41, 1.61)	0.81 (0.37, 1.77)	0.93 (0.37, 2.32)	0.95	0.99
No	1.00	0.90 (0.51, 1.59)	0.59 (0.31, 1.13)	0.30 (0.12, 0.74)	0.01	
*Alcohol drinking*						
Yes	1.00	0.63 (0.31, 1.30)	0.64 (0.29, 1.43)	0.39 (0.14, 1.18)	0.11	0.15
No	1.00	1.02 (0.59, 1.74)	0.62 (0.32, 1.18)	0.58 (0.26, 1.27)	0.11	
*BMI (kg/m*^*2*^*)*						
< 24	1.00	0.69 (0.37, 1.30)	0.66 (0.33, 1.32)	0.53 (0.20, 1.38)	0.21	0.87
24 ~ 28	1.00	0.88 (0.57, 1.34)	0.78 (0.48, 1.26)	0.59 (0.32, 1.09)	0.09	
> 28	1.00	1.27 (0.47, 3.41)	0.98 (0.31, 3.08)	1.12 (0.27, 4.64)	0.97	
*Hypertension*						
Yes	1.00	1.23 (0.70, 2.13)	0.62 (0.31, 1.24)	0.80 (0.36, 1.76)	0.33	0.85
No	1.00	0.45 (0.22, 0.93)	0.55 (0.26, 1.17)	0.18 (0.06, 0.57)	0.01	
Diabetes						
Yes	1.00	0.41 (0.15, 1.23)	0.48 (0.21, 1.15)	0.26 (0.17, 0.93)	0.67	0.89
No	1.00	0.83 (0.54, 1.29)	0.60 (0.36, 1.00)	0.46 (0.23, 0.89)	0.02	
*Myocardial infarction*						
Yes	1.00	0.68 (0.33, 1.25)	0.78 (0.37, 1.44)	0.59 (0.18, 1.24)	0.23	0.33
No	1.00	0.87 (0.56, 1.35)	0.66 (0.39, 1.09)	0.52 (0.27, 1.00)	0.04	

^1^Covariates: age (continuous), sex (men/woman), urbanization index (low, medium, high), education (primary, secondary, college/university), household income (low, middle, high), smoking status (yes/no), alcohol intake (yes/no), physical activity levels (light, moderate, vigorous), BMI (< 24, 24–28, or > 28 kg/m^2^), hypertension (yes/no), diabetes (yes/no), myocardial infarction (yes/no), medication use (yes/no), energy, whole grain, red meat, fruits, vegetables, saturated fat, polyunsaturated fat, cereal fiber, Na, K and cholesterol intakes (continuous), except for the stratifying variables per se.

^2^*P*_for trend_ values were calculated by modeling the median of each quartile-defined category as a continuous variable in the model.

^3^*P*_for interaction_ values were calculated using the likelihood-ratio test.

^4^In the stratified analysis, characteristics of participants at baseline were used for stratification and adjustment.

Three sensitivity analyses were conducted to assess the potential mediational factors and the robustness of the associations. When we repeated the analyses after excluding patients with hypertension at baseline, the direction and the association did not substantially change, with the HR of stroke for the highest quintile of calcium intake versus the lowest being 0.44 (95% CI 0.29–0.97, *P*_for trend_ = 0.04) (see Supplementary Table S1 online). Besides, when we used the non-adjusted person time, the results remained the same (HR: 0.53, 95% CI 0.29–0.97, *P*_for trend_ = 0.03) (see Supplementary Table S2 online). Finally, conducting dyslipidemia (HR: 0.53, 95% CI 0.29–96, *P*_for trend_ = 0.03) further adjustment did not materially change the association (see Supplementary Table S3 online).

## Discussion

In this large prospective cohort study among the Chinese population, we discovered higher intake of dietary calcium was significantly associated with lower stroke risk. Stratified analysis displayed a strong inverse association between calcium intake and stroke risk among men. No significant association was observed for either dietary magnesium or phosphorus intake with stroke risk.

Prior epidemiological studies have reported different results concerning the relationship between calcium intake and stroke risk. A large prospective cohort study, including 36,094 subjects, observed that calcium intake did not relate to stroke risk in Europe^11^. Likewise, in a prospective study in American adults, Ascherio et al.^13^ found no association between dietary calcium intake and stroke risk. The result is consistent with the findings of Sluijs et al.^27^, who indicated that increased calcium intake was not associated with a decreased risk of stroke in the EPIC-NL Study. However, another large prospective study reported conflicting results with calcium intake being inversely associated with incident stroke^10,28,29^. Hiroyasu et al. using the Nurses' Health Study data, noted that the increase in risk was limited to the lowest calcium intake quintile. Intakes ≥ 600 mg/d did not seem to reduce stroke risk further^10^. Also, in Japanese adults (range of intake 233 to 753 mg/d), participants in the highest quintile of dietary calcium intake had a 30% lower risk of stroke than the lowest quintile^30^. Moreover, Larsson, S. C. et al. conducted a meta-analysis of 11 prospective research, finding that dietary calcium was inversely related to stroke risk in Asian populations and other populations with low to moderate calcium intakes^31^. Similarly, a significant inverse association between calcium consumption (range of intake 210 to 594 mg/d) and stroke risk was found in our study.

A possible explanation for the inconsistent results is that calcium intake derived from the diet differed substantially between these studies. It is well-established that the Asian have much lower calcium intake than the European and American populations, whether in the nation, age, and gender^32^. For example, the Japanese median calcium intake in the highest category (642 mg/d), which was reported in the Japan Public Health Center (JPHC) study^30^, was lower than that of in the lowest category from a study of Finnish men (876 mg/d)^33^. Moreover, the primary sources of dietary calcium are different across different populations. In Europe and America, dairy products are the primary source of calcium. In contrast, non-dairy foods, such as vegetables, soy products, and grains, are the main contributors to calcium in Asia. A study reported that some nutrients in non-dairy products, such as phytic acid and uronic acid residues, would combine with calcium to form insoluble substances that inhibit the body's absorption of calcium^34^. The confounding effect of these nutrients likely results in either attenuation of the association or a spurious unassociated among calcium consumption and stroke risk. since they can regulate vascular calcification and endothelial dysfunction.

The biological mechanisms underlying the putative effects of calcium on stroke risk deserve elucidation. Decreased dietary calcium may induce calcium depletion from all membrane storage sites, leading to instability of the vascular smooth muscle cell membrane^35^, which may trigger vasoconstriction and, possibly, elevated blood pressures and stroke. In addition to the possible direct action on smooth muscle cell contraction, calcium may also have indirect effects by modifying the responsiveness to or regulating the synthesis of vasoactive mediators, including angiotensin II and nitric oxide^36^. Kawasaki et al.^37^discovered that vascular sensitivity to angiotensin II decreased evidently after the supplementation of calcium. Moreover, some randomized controlled trials proposed that calcium supplementation reduced vascular smooth muscle contractility, possibly increasing nitric oxide synthesis^38,39^. Besides, some studies also have demonstrated that lower calcium intake may cause vasoconstriction either through stimulation of parathyroid hormone (PTH) or renin release, resulting in further elevated blood pressure^40,41^. Moreover, work regarding calcium's role suggests that calcium could induce artery relaxation and modulate local blood pressure via calcium receptors^42^. Except for the hypotensive effect, calcium can reduce platelet aggregation and lower plasma total cholesterol levels via forming insoluble complexes with fatty acids and reducing the absorption of fatty acids, which may lower the risk of stroke^43^.

An impressive result was observed in our study that dietary calcium consumption was related only to a reduced risk of stroke in men but not women. The difference may be explained by estrogen fluctuation; ovarian hormone production declines in a premenopausal phase. The decreased level of circulating estrogen aggravates the detrimental effect of stroke and counterbalances the beneficial role of calcium in postmenopausal women^44^. A recent randomized trial of 36,282 postmenopausal women over 7 years also revealed that calcium supplements combined with vitamin D neither decreased nor increased the risk of stroke^45^. On the other hand, this discrepancy also could be due to differences in the remaining stroke risk factors among sexes. Compared with men, women are relatively inaccessible to environmental risk factors, such as cigarette smoking, making blood vessels spasm due to harmful substances in the tobacco, and increasing blood coagulability and viscosity or acceleration arteriosclerosis associated. Since men are more likely exposed to environmental risk factors than women, protective factors are more likely to be observed in riskier environments. Hence, our study found a stronger association between male intake and stroke, not in women.

As for magnesium intake with stroke, no significant association was found in the Nurses' Health Study^10^ or the Swedish Mammography Cohort^30^. Similarly, in some randomized trials, magnesium supplementation did not reduce blood pressure in normotensive volunteers^46^. A high magnesium diet was observed to increase rather than decrease the risk of stroke in hypertensive rats^47^. Our study was consistent with previous studies in which the relationship between dietary magnesium intake and stroke. In both animal models and clinical studies, phosphorus intake affects the release of PTH and fibroblast growth factor-23 (FGF-23), both of which may increase oxidative stress in endothelial cells, lead to arterial calcification^48^ and endothelial dysfunction^49^. According to a recent opinion in European Heart Journal, excess dietary phosphorus intake contributes to cardiovascular disease by raising serum phosphorus concentrations^50^ and indirectly support a preventive impact of phosphorus on the risk of stroke. However, no prominent relationship between dietary phosphorus and stroke risk was observed in our study. Given the sparse evidence, more compelling researches are necessary to reveal the association between dietary phosphorus intake and stroke.

As far as we know, there have been no studies on dietary calcium, magnesium, and phosphorus intake with stroke risk in the Chinese population. The major strengths of the present study include its large sample size and prospective design, which reduced the possibility of recall and selection biases. In addition, dietary data were collected before the occurrence of the disease so that disease status could not influence the self-report of diet. Furthermore, by calculating the cumulative average of dietary intakes, we minimized the measurement errors caused by a diet change over time and considered eating behavior changes.

Some limitations of the present study are worth discussion as well. Firstly, the dietary assessment was a 24-h dietary recall, which only reflects the short-term dietary situation. However, the approach used was validated, which does not cause fatal to the significance of the study, to estimate individual dietary intake, whether in extensive national dietary surveys or small studies. Secondly, the information on nutritional supplements of each individual was not collected. Therefore, we were unable to evaluate the effect of supplements. Nevertheless, according to a report based on the China National Nutrition and Health Survey in 2002, only 3.4% of adults aged 45 to 59 years and 5.0% of adults aged older than 60 years took calcium supplements^51^. Thirdly, stroke events, diabetes events, and myocardial infarction events in this database are based on self-reports. There is no cause of death investigation report; some inaccuracies in the data may exist due to the lack of information on stroke death. Fourthly, we considered the influence of cancer on stroke in our model analysis. However, the CHNS database only started to study cancer in the population since 2009. Without baseline data on cancer in the population, we did not include them in the model. We only included dyslipidemia as a sensitivity analysis (see Supplementary Table S3, S4 online). Fifthly, we considered the effects of hormone therapy (postmenopausal women) on stroke. But in the CHNS survey, the questionnaire did not provide information about hormone therapy in postmenopausal women, so we didn't include them in the model. Finally, residual confounding from imprecisely or unmeasured risk factors could not be excluded entirely, which may hinder causal inference based on these observations, though we controlled other confounding variables for stroke. However, we carefully controlled a broad range of potential covariates, and the stratification analyses and sensitivity analyses did not appreciably alter our results.

In conclusion, this prospective cohort study in the Chinese population demonstrated that dietary calcium intake was inversely associated with stroke risk, especially in the man. According to our present study, increasing dietary calcium consumption may be an important part of diet interventions to reduce stroke risk. Our findings on the effect of calcium intake on stroke have important clinical and public health implications and provide strong support for current dietary guidelines. No statistical association of dietary magnesium and phosphorus intakes on stroke risk was observed. Further research will need a larger sample size to verify this relationship between calcium intake and stroke in women.

## Methods

### Study population

The target subjects of the present study were participants in the CHNS, which is an ongoing open, prospective household-based study conducted since 1989 to investigate health risk factors, nutritional status, sociological, demographic transformation, and health-related outcomes of the Chinese population^16^. CHNS applies a multistage random-cluster sampling technique to select representative individuals from twelve provinces across China. The CHNS cohort includes new household formation and replacement communities and households; all household members are studied. Furthermore, in-depth community data are collected. Detailed information about the sampling procedure on the cohort has been reported elsewhere, and they are used jointly and separately in hundreds of studies^17–19^. This study was reviewed and authorized by Institutional Review Boards of the University of North Carolina at Chapel Hill and the National Institute of Nutrition and Food Safety, China Center for Disease Control and Prevention. Due to its excellent properties such as stratified random sampling, large sample, and panel data, CHNS data has become the basic data of this study. All methods were performed under their relevant guidelines and regulations. For investigating the association of dietary calcium, magnesium, and phosphorus with stroke, the data from CHNS surveys during 2004–2011 were used because it has provided more accurate dietary data since 2004. All the participants were restricted to those aged 45–79 years to ensure a relatively high stroke incidence, who had complete data about socio-economic, anthropometric, demographic, and three-day, 24 h dietary recalls. We excluded pregnant or lactating women, those who had implausible energy intakes (< 800 kilocalories (kcal) per day or > 6000 kcal for men and < 600 kcal or > 4000 kcal for women^20^) or only participated in one survey in the four surveys from 2004 to 2011. Therefore, the current analysis consisted of 6411 participants who were available for the analysis. All subjects supplied their informed consent.

### Dietary intake

Data on dietary intake was collected by three consecutive 24-h dietary recall at the individual level coupled with conversion of cooking ingredients (such as oils and condiments) at the household level acquired by the food inventory weighing method over the same 3-d duration^21^. The average daily intake of calcium, magnesium, or phosphorus was calculated by multiplying the ingestion of each food item by the nutrient content of standard portion size of 100 g based on the Food Composition Tables, then summing the intake levels of nutrients throughout all food items.

### Outcome measurement

The outcome of the present study was the incident stroke that occurred during the follow-up. Stroke was defined based on a self-report of diagnosis by a physician in public hospitals above the county level. The participants should provide a certificate of stroke diagnosis. Investigators need to verify the patient's diagnosis of proof and inquiry the questions ‘Has a doctor ever given you the diagnosis of stroke or transient ischemic attack? How old were you when you were first diagnosed with a stroke or transient ischemic attack?’. For participants who provided inconsistent answers during the follow-up, the first recorded stroke event was adopted to limit recall bias.

### Assessments of covariates

Questionnaire-based interviews collected basic information of participants on age, sex, urbanization index, education, annual household income, smoking status, alcohol intake, physical activity, body weight, hypertension, diabetes, myocardial infarction, and medication use. The urbanization index determined in individuals is based on the community in which they live. At the community level, the CHNS measures the urbanization index in 12 dimensions: population density, economic activity, traditional markets, modern markets, transport infrastructure, health, communications, housing, education, diversity, health infrastructure, and social services. The in-depth community contextual measures in the CHNS database enabled us to create an indicator of the major scale of modernization covering all 288 communities. Urbanization index and household income were divided into tertiles (low, middle, or high). Educational attainment was categorized into three levels (primary, secondary, or college/university). Tobacco and alcohol consumption were both dichotomized as never or ever. Physical activity was separated into three levels (light, moderate, or vigorous). Physical activity, including four main areas: housework, occupation, transportation, and leisure activity, was evaluated by metabolic equivalent task hours for each week (MET-h/week), which was calculated by multiplying the amount of time spent in each activity with its assigned metabolic equivalent intensity according to the Compendium of Physical Activities, and then summing the metabolic equivalent of each activity^22^. Weight and height were obtained by measuring. After removing shoes and heavier clothing, participants were measured to an accuracy of 0.1 cm in height and 0.1 kg in weight. Body mass index (BMI) was calculated as weight in kilograms divided by squared height in meters (kg/m^2^). Blood pressure was measured in a quiet environment. After emptying their bladders, the participants sat quietly for more than five minutes. Their blood pressure was measured using a calibrated mercury sphygmomanometer by a trained health nurse following a standardized procedure. The participants placed their right arm in a cuff airbag, measured at least 1 min between recordings. We defined hypertension patients who have a mean of SBP ≥ 140 mmHg, a mean of DBP ≥ 90 mmHg, or taking antihypertensive medication according to the guidelines for preventing and treating hypertension in China (2010)^23^. Diabetes and myocardial infarction were defined based on self-report of diagnosis by a physician in public hospitals above the county level. The participants should provide the certificate of diabetes or myocardial infarction. Investigators need to verify the patient's diagnosis of proof and inquiry the questions ‘Has a doctor ever given you the diagnosis of diabetes? Has a doctor ever given you the diagnosis of myocardial infarction?’. Medication use was defined based on the participants’ self-report, in which investigators need to ask the participants the following questions: ‘Are you currently taking anti-hypertension drugs? Did you use the oral medicine, injection of insulin, Chinese traditional medicine, or home remedies to treat diabetes?’ After a continuous fasting period of at least 10 h, blood samples were collected from all eligible participants. According to the guidelines for the prevention and treatment of blood lipids in Chinese adults (2006), participants who met at least one of the following criteria: (1) TC ≥ 6.22 mmol/L, (2) HDL-C < 1.04 mmol/L, (3) LDL-C ≥ 4.14 mmol/L, (4) TG ≥ 2.26 mmol/L were defined as dyslipidemia patients. We also considered other dietary covariates, such as total dietary energy, energy, whole grain, red meat, fruits, vegetables, saturated fatty acid, polyunsaturated fatty acid, cereal fiber, Na, K, and cholesterol intake, as the surrogates to control potential dietary confounding.

### Statistical analysis

The cumulative average intake of calcium, magnesium, and phosphorus was calculated to minimize within-person variation and thus gain the best estimate of the long-term intake. Person-years of follow-up for each participant were calculated as the interval between the date of finishing of the baseline questionnaire and the end of follow-up (the date of the last visit or the visit of stroke diagnosis), whichever came first. The incidence rate was calculated by dividing the number of cases by person-years of follow-up. The multivariate Cox proportional hazards models were used to estimate the hazard ratios (HR) and 95% confidence intervals (95% CI) of developing stroke according to dietary calcium, magnesium, and phosphorus intake using the lowest quartile as the reference group. In the Cox proportional hazards models, the p for trend is performed by modeling the median of the categories defined by each quartile as a continuous variable.

We first performed statistical interaction tests between dietary intakes of calcium, magnesium, phosphorus, and other baseline characteristics (Table 1). We used the Kolmogorov-Sminov test to test the normality of all variables. We outlined the baseline characteristics of the participants. We used the t-test for the continuous variables with normal distribution to test for differences and trends between groups. The non-parametric test is applied to the continuous variables with non-normal distribution, and the chi-square test is used for the classified variables. Then, we categorize the three-day average intakes of calcium, magnesium, phosphorus into four levels. We constructed three sequential models to estimate the effect of calcium, magnesium, phosphorus intakes on stroke. Model 1 presented the age and sex-adjusted results. Model 2 further added baseline sociodemographic and lifestyle factors such as urbanization index, education, household income, smoking status, alcohol intake, physical activity, BMI, hypertension, diabetes, myocardial infarction, and medication use into the multivariable analysis. Model 3 additionally adjusted for dietary factors including energy (kcal/d), whole grain (g/d), red meat (g/d), fruits (g/d), vegetables (g/d), saturated fatty acid (g/d), polyunsaturated fatty acid (g/d), cereal fiber (g/d), Na (mg/d), K (mg/d) and cholesterol (g/d)^24–26^. To further analyze which factors would affect the relationship between the exposure and outcome, we conducted stratified analyses by age at baseline (< 60 and ≥ 60 years), sex (man and woman), smoking (never and ever), drinking (never and ever), BMI (< 24, 24–28 and > 28 kg/m^2^), hypertension, diabetes, and Myocardial infarction. The likelihood ratio test was used to assess the interactions of cross-product terms. The proportional hazards assumption for dietary calcium, magnesium, phosphorus Intakes, and other covariates was checked by Schoenfeld’s residuals, and no violation was found (P > 0.05). Besides, we performed sensitivity analyses to verify the robustness of the primary results. First, we excluded cases diagnosed with hypertension at baseline because these participants were more likely to change their dietary habits after perceiving risk. Second, we repeated the analysis using the non-adjusted person-time from the date of the first visit to the time of the most recent visit of stroke diagnosis or to the end of follow-up, whichever came first. Third, we included dyslipidemia as a sensitivity analysis, which confirmed the robustness of our findings to some degree. Tests for trends across dietary intake categories were conducted by modeling the median value in each quartile as a continuous variable in each model. All statistical analyses were performed with STATA version 14.0. All *P*-values were two-sided, and *P* < 0.05 was considered to statistical significance.

## Supplementary Information

Supplementary Tables.
