# Supplementary material for: Dietary calcium, magnesium, and phosphorus intakes and risk of stroke in Chinese adults
Source: Sci Rep. 2021 May 28;11:11270. doi: 10.1038/s41598-021-90388-z (PMC8163833; doi:10.1038/s41598-021-90388-z)
Supplement: Supplementary file 1 — Supplementary Tables. [file 41598_2021_90388_MOESM1_ESM.pdf]

Supplementary Information

Dietary Calcium, Magnesium, and Phosphorus Intakes and Risk of Stroke in Chinese Adults

Hai-Lu Zhu [1, 2, 3, †], Yan Liu [1, 2, †], Jian Zhang [3, †], Ming-Xu Wang [3], Hong Jiang [3], Fang Guo [3,4], Ming Li [5], Fei-Fei Qi [3, *], Xiao-Hong Liu [1, *] and Le Ma [3, *]

[1] The First Affiliated Hospital, Xi'an Jiaotong University Health Science Center, Xi'an 710061, People's Republic of China;

[2] Key Laboratory of Shaanxi Province for Craniofacial Precision Medicine Research, College of Stomatology, Xi'an Jiaotong University, Xi'an, China;

[3] School of Public Health, Xi'an Jiaotong University Health Science Center, Xi'an 710061, People's Republic of China;

[4] School of Public Health, University of Hong Kong, Pok Fu Lam, Hong Kong Special Administrative Region 999077, People's Republic of China

[5] Center for Population Health Research, Division of Health Sciences, University of South Australia, Adelaide 5000, South Australia, Australia

[*] Corresponding author. Fei-Fei Qi, Email: qifeifei@mail.xjtu.edu.cn. Xiao-Hong Liu, Email: liuxiaoh@mail.xjtu.edu.cn. Le Ma, Email: male@mail.xjtu.edu.cn

[†] These authors contributed equally to this work.

Supplementary Table S1. Hazard ratios (95% confidence intervals) of stroke according to quartiles of dietary calcium, magnesium, and phosphorus intakes

	Dietary nutrition intake				P for trend[1]
	Q 1 (low)	Q 2	Q 3	Q 4 (high)	
Calcium					
Median intake (mg/d)	222	322	412	677	
Person-years	5161	5918	5967	5215	
Stroke cases (n)	37	33	31	20	
Model 1	1.00	0.74 (0.46, 1.20)	0.70 (0.41, 1.13)	0.52 (0.30, 0.91)	0.03
Model 2	1.00	0.76 (0.60, 1.35)	0.75 (0.45, 1.25)	0.52 (0.29, 0.93)	0.03
Model 3	1.00	0.78 (0.46, 1.30)	0.76 (0.43, 1.33)	0.44 (0.21, 0.95)	0.04
Magnesium					
Median intake (mg/d)	200	265	320	443	
Person-years	4742	5870	5925	5734	
Stroke cases (n)	33	26	27	35	
Model 1	1.00	0.67 (0.40, 1.13)	0.75 (0.44, 1.26)	0.88 (0.54, 1.45)	0.36
Model 2	1.00	0.75 (0.43, 1.30)	0.82 (0.47, 1.43)	1.11 (0.66, 1.87)	0.89
Model 3	1.00	0.89 (0.50, 1.60)	1.02 (0.54, 1.93)	1.32 (0.62, 2.82)	0.91
Phosphorus					
Median intake (mg/d)	668	875	1043	1393	
Person-years	4977	5880	5889	5525	
Stroke cases (n)	37	31	19	34	
Model 1	1.00	0.75 (0.46, 1.22)	0.26 (0.26, 0.79)	0.83 (0.50, 1.35)	0.06
Model 2	1.00	0.83 (0.50, 1.38)	0.46 (0.25, 0.85)	0.97 (0.58, 1.64)	0.43
Model 3	1.00	1.05 (0.60, 1.85)	0.65 (0.32, 1.34)	1.46 (0.63, 3.39)	0.90

[1]Tests for trend were conducted by modeling the median of each quartile-defined category as a continuous variable in Cox proportional hazards models.

Model 1, adjusted for age and sex.

Model 2, further adjusted for urbanization index, education, household income, smoking status, alcohol intake, physical activity, BMI, hypertension, diabetes, myocardial infarction and medication use based on model 1.

Model 3, further adjusted for energy, whole grain, red meat, fruits, vegetables, saturated fat, polyunsaturated fat, cereal fiber, Na, K, and cholesterol intakes based on model 2.

Supplementary Table S2. Hazard ratios (95% confidence intervals) of stroke according to quartiles of dietary calcium, magnesium, and phosphorus intakes

	Dietary nutrition intake				P for trend[1]
	Q 1 (low)	Q 2	Q 3	Q 4 (high)	
Calcium					
Median intake (mg/d)	222	321	413	677	
Person-years	7657	8399	8467	7711	
Stroke cases (n)	52	51	43	33	
Model 1	1.00	0.87 (0.59, 1.28)	0.72 (0.48, 1.08)	0.59 (0.38, 0.92)	0.02
Model 2	1.00	0.90 (0.60, 1.35)	0.66 (0.42, 1.02)	0.59 (0.37, 0.94)	0.02
Model 3	1.00	0.88 (0.58, 1.35)	0.66 (0.41, 1.08)	0.53 (0.29, 0.97)	0.03
Magnesium					
Median intake (mg/d)	200	265	320	443	
Person-years	7173	8309	8496	8046	
Stroke cases (n)	53	39	41	46	
Model 1	1.00	0.66 (0.43, 1.00)	0.70 (0.46, 1.06)	0.77 (0.51, 1.17)	0.43
Model 2	1.00	0.70 (0.44, 1.08)	0.72 (0.46, 1.12)	0.96 (0.63, 1.48)	0.84
Model 3	1.00	0.76 (0.48, 1.22)	0.79 (0.47, 1.32)	0.98 (0.51, 1.86)	0.89
Phosphorus					
Median intake (mg/d)	667	875	1043	1393	
Person-years	7441	8302	8415	7866	
Stroke cases (n)	61	46	28	44	
Model 1	1.00	0.70 (0.48, 1.04)	0.41 (0.26, 0.64)	0.67 (0.45, 1.01)	0.05
Model 2	1.00	0.75 (0.50, 1.12)	0.41 (0.25, 0.66)	0.80 (0.52, 1.24)	0.26
Model 3	1.00	0.84 (0.53, 1.32)	0.48 (0.27, 0.85)	0.94 (0.61, 1.90)	0.82

[1]Tests for trend were conducted by modeling the median of each quartile-defined category as a continuous variable in Cox proportional hazards models.

Model 1, adjusted for age, sex.

Model 2, further adjusted for urbanization index, education, household income, smoking status, alcohol intake, physical activity, BMI, hypertension, diabetes, myocardial infarction and medication use based on model 1.

Model 3, further adjusted for energy, whole grain, red meat, fruits, vegetables, saturated fat, polyunsaturated fat, cereal fiber, Na, K, and cholesterol intakes based on model 2.

Supplementary Table S3. Hazard ratios (95% confidence intervals) of stroke according to quartiles of dietary calcium, magnesium, and phosphorus intakes

	Dietary nutrition intake				P for trend[1]
	Q 1 (low)	Q 2	Q 3	Q 4 (high)	
Calcium					
Median intake (mg/d)	222	321	413	677	
Person-years	5748	6705	6770	6021	
Stroke cases (n)	52	51	43	33	
Model 1	1.00	0.87 (0.59, 1.28)	0.72 (0.48, 1.08)	0.59 (0.38, 0.92)	0.02
Model 2	1.00	0.90 (0.60, 1.35)	0.65 (0.42, 1.02)	0.59 (0.37, 0.94)	0.02
Model 3	1.00	0.88 (0.57, 1.34)	0.66 (0.41, 1.08)	0.53 (0.29, 0.96)	0.03
Magnesium					
Median intake (mg/d)	200	265	320	443	
Person-years	5466	6625	6798	6356	
Stroke cases (n)	53	39	41	46	
Model 1	1.00	0.66 (0.43, 1.00)	0.70 (0.46, 1.06)	0.77 (0.51, 1.17)	0.43
Model 2	1.00	0.69 (0.44, 1.08)	0.72 (0.46, 1.12)	0.96 (0.62, 1.48)	0.84
Model 3	1.00	0.76 (0.48, 1.22)	0.79 (0.47, 1.32)	0.98 (0.51, 1.86)	0.90
Phosphorus					
Median intake (mg/d)	667	875	1043	1393	
Person-years	5732	6618	6722	6172	
Stroke cases (n)	61	46	28	44	
Model 1	1.00	0.70 (0.48, 1.04)	0.41 (0.26, 0.64)	0.67 (0.45, 1.01)	0.05
Model 2	1.00	0.75 (0.50, 1.12)	0.41 (0.25, 0.66)	0.80 (0.52, 1.24)	0.26
Model 3	1.00	0.84 (0.53, 1.32)	0.48 (0.27, 0.85)	0.94 (0.61, 1.90)	0.81

[1]Tests for trend were conducted by modeling the median of each quartile-defined category as a continuous variable in Cox proportional hazards models.

Model 1, adjusted for age and sex.

Model 2, further adjusted for urbanization index, education, household income, smoking status, alcohol intake, physical activity, BMI, hypertension, diabetes, myocardial infarction, medication use, and dyslipidemia based on model 1.

Model 3, further adjusted for energy, whole grain, red meat, fruits, vegetables, saturated fat, polyunsaturated fat, cereal fiber, Na, K, and cholesterol intakes based on model 2.

Supplementary Table S4. Stratified hazard ratios (95% confidence intervals)[1] of stroke according to quartiles of dietary calcium intake by various characteristics of participants.

	Dietary calcium intake				$P_{\text{for trend}}$[2]	$P_{\text{for interaction}}$[3]
	Q 1 (low)	Q 2	Q 3	Q 4 (high)		
Sex[4]						
Man	1.00	0.53 (0.31, 0.90)	0.52 (0.28, 0.95)	0.32 (0.14, 0.73)	0.01	0.02
Women	1.00	2.00 (0.94, 4.22)	0.94 (0.38, 2.37)	1.22 (0.45, 3.32)	0.87	
Age (years)						
<60	1.00	0.67 (0.37, 1.27)	0.59 (0.28, 1.24)	0.28 (0.09, 0.80)	0.02	0.06
≥60	1.00	1.09 (0.59, 2.00)	0.72 (0.36, 1.47)	0.86 (0.37, 1.97)	0.57	
Cigarette smoking						
yes	1.00	0.79 (0.40, 1.56)	0.77 (0.35, 1.68)	0.86 (0.34, 2.19)	0.86	0.89
no	1.00	0.90 (0.51, 1.59)	0.59 (0.31, 1.13)	0.30 (0.12, 0.72)	0.01	
Alcohol drinking						
yes	1.00	0.64 (0.31, 1.32)	0.65 (0.29, 1.46)	0.38 (0.13, 1.13)	0.11	0.10
no	1.00	1.01 (0.59, 1.74)	0.60 (0.31, 1.15)	0.56 (0.25, 1.23)	0.09	
BMI (kg/m^2)						
<24	1.00	0.70 (0.37, 1.30)	0.66 (0.33, 1.32)	0.53 (0.20, 1.38)	0.21	0.83
24~28	1.00	0.87 (0.57, 1.33)	0.76 (0.48,1.23)	0.57 (0.31, 1.04)	0.06	
>28	1.00	1.25 (0.46, 3.35)	0.95 (0.30, 2.97)	1.06 (0.26, 4.36)	0.96	
Hypertension						
yes	1.00	1.23 (0.70, 2.13)	0.61 (0.30, 1.22)	0.75 (0.34, 1.66)	0.26	0.73
no	1.00	0.44 (0.22, 0.92)	0.55 (0.25, 1.17)	0.19 (0.06, 0.59)	0.01	
Diabetes						
yes	1.00	0.40 (0.14, 1.23)	0.50 (0.23, 1.16)	0.26 (0.17, 0.93)	0.58	0.78
no	1.00	0.83 (0.54, 1.29)	0.60 (0.36, 1.00)	0.44 (0.23, 0.87)	0.01	
Myocardial infarction						
yes	1.00	0.67 (0.33, 1.29)	0.78 (0.37, 1.44)	0.60 (0.19, 1.27)	0.20	0.25
no	1.00	0.86 (0.55, 1.23)	0.66 (0.39, 1.09)	0.50 (0.24, 0.96)	0.02	

[1]Covariates: age (continuous), sex (men/woman), urbanization index (low, medium, high), education (primary, secondary, college/university), household income (low, middle, high), smoking status (yes/no), alcohol intake (yes/no), physical activity levels (light, moderate, vigorous), BMI (< 24, 24-28, or > 28 kg/m^2), hypertension (yes/no), diabetes (yes/no), myocardial infarct (yes/no), dyslipidemia (yes/no), medication use (yes/no), energy, whole grain, red meat, fruits, vegetables, saturated fat, polyunsaturated fat, cereal fiber, Na, K and cholesterol intakes (continuous), except for the stratifying variables per se.

[2]$P_{\text{for trend}}$ values were calculated by modeling the median of each quartile-defined category as a continuous variable in the model.

[3]$P_{\text{for interaction}}$ values were calculated using the likelihood-ratio test.

[4]In the stratified analysis, characteristics of participants at baseline were used for stratification and adjustment
